# Quantification of structural changes of UHMWPE components in total joint replacements

**DOI:** 10.1186/1471-2474-15-109

**Published:** 2014-03-28

**Authors:** Petr Fulin, David Pokorny, Miroslav Slouf, Martina Nevoralova, Tatana Vackova, Jiri Dybal, Jan Pilar

**Affiliations:** 1 Department of Orthopaedics, First Faculty of Medicine, Charles University in Prague, V Úvalu 84, Prague 5 15006, Czech Republic; 2 University Hospital Motol, V Úvalu 84, Prague 5 15006, Czech Republic; 3 Institute of Macromolecular Chemistry, Academy of Sciences of the Czech Republic, Heyrovsky Sq. 2, 162 06, Prague 6, Czech Republic

**Keywords:** UHMWPE, Oxidative damage, Residual radicals, Infrared spectroscopy, Electron spin resonance

## Abstract

**Background:**

At present time the number of implantations of joint replacements as well as their revisions increases. Higher demands are required on the quality and longevity of implants. The aim of this work was to determine the degree of oxidative degradation and the amount of free/residual radicals in selected ultra-high molecular weight polyethylene (UHMWPE) components of the joint replacements and demonstrate that the measured values are closely connected with quality and lifetime of the polymer components.

**Methods:**

We tested both new (4 samples) and explanted (4 samples) UHMWPE polymers for total joint replacements. The samples were characterized by infrared spectroscopy (IR), electron spin resonance (ESR) and microhardness (MH) test. The IR measurements yielded the values of oxidation index and trans-vinylene index. The ESR measurements gave the free radicals concentration.

**Results:**

In the group of new polyethylene components, we found oxidation index values ranging from 0.00-0.03 to 0.24. The trans-vinylene index values ranged from 0.044 to 0.080. The value of free radical concentration was zero in virgin and also in sample of Beznoska Company and non-zero in the other samples. In the group of explanted components, the measured values were associated with their history, micromechanical properties and performance in vivo.

**Conclusions:**

We demonstrated that measuring of oxidative damage may help the orthopaedic surgeon in estimating the quality of UHMWPE replacement component and thus radically to avoid early joint replacement failure due to worse polyethylene quality.

## Background

Ultra-high molecular weight polyethylene (UHMWPE) is and undoubtedly will remain for a number of years the most frequently used material for the manufacture of bearing components of large joint replacements. This polymer exhibits suitable properties for given application: excellent bio-compatibility, sufficient mechanical properties (toughness, resistance to impact etc.), and above-the-average tribological properties (low friction coefficient and high resistance to wear).

Application of UHMWPE is associated with several problems and complications. As UHMWPE liners represent the most loaded part of the endoprosthesis, a high percentage of joint replacement failures were actually associated with residual radicals in UHMWPE components. Nowadays, however, the UHMWPE modification procedures were improved [1-4] and the total joint prostheses fail mainly due to aseptic loosening, infection, or instability [5]. Based on data available in literature [6,7] as well as on our own experience [8-11], it may be concluded that there exist two principal material-related reasons of UHMWPE failure: ***wear*** and ***oxidative damage.*** It is worth noting that the two phenomena are closely connected: the oxidative degradation leads to the decrease in mechanical properties including wear resistance [3,4].

The process of polyethylene **wear** has been explained comprehensibly and in detail in literature [12-14]. In contrast, only a minimum of information is available in orthopaedic literature regarding the principles and mechanisms of **oxidative damage**. In fact, oxidation of UHMWPE fundamentally alters key properties, including the aforementioned resistance to wear, which affects the lifespan of the given joint component [15].

Cleavage of the chemical bond within the polyethylene molecule chain may be due to several chemical, physical or mechanical reasons. In the case of components manufacturing from UHMWPE, interaction with gamma radiation is a frequent cause of structural changes, namely generation of vinylene groups. The aforementioned radiation is used during the manufacture of UHMWPE based joint replacements to cross-link the polymer [16] or to sterilise it [2,3]. Interaction of radiation with the polymers leads to the homolytic cleavage of polymer bonds. Alkyl radicals are the primary products of this process [17]. However, the alkyl radicals are highly reactive and rapidly undergo further reactions. The first practically significant reaction of alkyl radicals is the cleavage of hydrogen and the genesis of a double bond (C = C), so-called *trans-vinylene bond*. It follows logically that the number of C = C bonds is to a certain extent proportional to the amount of generated radicals, which is in turn proportional to the radiation dose used [11]. Moreover, a higher dose corresponds (under the given conditions) to a higher degree of polymer cross-linking. It may thus be summarised that by measuring the concentration of trans-vinylene bonds within a sample it is possible to indirectly estimate the radiation dose used for the given type of UHMWPE as well as the degree of cross-linking of the modified polyethylene [4,11]. Another important reaction of alkyl radicals, which from the aspect of joint replacement lifespan is by far most significant, involves their combination and reaction with oxygen molecules. Oxygen molecules are partially contained in the polymer, where they diffuse from the surrounding environment, i.e. from the atmosphere or the inner environment of tissues and fluids. It is well known from literature that the reaction of alkyl radicals with oxygen is cyclic [17]. It may thus be concluded that a single radical may destroy many molecules of the polymer before it is destroyed by some random reaction. As oxidative degradation leads to the cleavage of polymer chains, the resulting UHMWPE structure and properties start to resemble standard (lower molecular weight) HDPE. Increased oxidation of the material thus leads to the cleavage of molecules, which in turn results in the deterioration of mechanical properties and this may lead to decreased resistance to wear or even to mechanical failure of the component.

For the orthopaedic surgeons, it is not easy to access comprehensible information concerning material properties and, as a result, the clinical specialists do not have much opportunity to form an objective, independent assessment of the quality of joint replacements available on the market. This makes the choice of joint replacement components with the best expected in vivo lifespan difficult.

The aim of this work is to report our results of polyethylene oxidative damage measurements and show that such a polyethylene component testing should help objectively assess the quality of UHMWPE components from various manufacturers. The methods used are applicable not only for testing new samples but also for testing and feedback evaluation of the quality of explanted joint replacement components.

## Methods

We tested two groups of samples. The first included various types of new (unused) UHMWPE joint replacement liners purchased directly from the manufacturer. All samples were purchased at the same time and stored under the same conditions in order to minimise the influence of storage on the properties of the material. Virgin, non-modified, medical grade UHMWPE (sample designated PE0, purchased directly from the manufacturer, MediTECH Germany) was added to these samples for comparison. The rest of the tested materials represented highly cross-linked types of UHMWPE from foremost American manufacturers (samples designated PE1 and PE2; processing details of PE1 - sequentially irradiated and annealed to total dose of 75 kGy, sterilized by ethylene oxide; processing details of PE2 – one step irradiated to 50 kGy, remelted, and sterilized by gamma irradiation using barrier packaging) and cross-linked UHMWPE manufactured in accordance with the process patented by the Institute of Macromolecular Chemistry of the Academy of Sciences of the Czech Republic (sample denoted as PE3; processing details: gamma irradiation to 75 kGy, remelting, ethylene oxide sterilization) and used in the joint replacements manufactured by Beznoska, Kladno, Czech Republic. These were materials that were available on the European market since 2007.

The second group of samples consisted of a group of 4 explanted polyethylene components. These included 3 hip joint replacement cups and 1 knee replacement plateau (Table 1), which were removed during revision surgery due to aseptic loosening. Sampling was performed at the Department of Orthopaedics, First Faculty of Medicine, Charles University in Prague and University Hospital Motol during years 2006–2007. The explanted polyethylene components were stored at a temperature below 0°C until their transport to the IMC ASCR for infrared spectroscopy (IR) and electron spin resonance (ESR) analyses.

**Table 1:** Various types of UHMWPE, explanted from joint replacements that failed due to aseptic loosening

**Sample**	**Joint**	**Manufacturer**	**Processing conditions**	**Year of implantation**	**Years in vivo**
L1	hip	DePuy / J&J	Noncrosslinked; gamma sterilized	1996	11
L2	knee	Walter Motorlet	Noncrosslinked; gamma sterilized	1987	20
L3	hip	Beznoska sro	Noncrosslinked; gamma sterilized	1991	16
L4	hip	Beznoska sro	Noncrosslinked; gamma sterilized	1996	10

**Figure 1:**
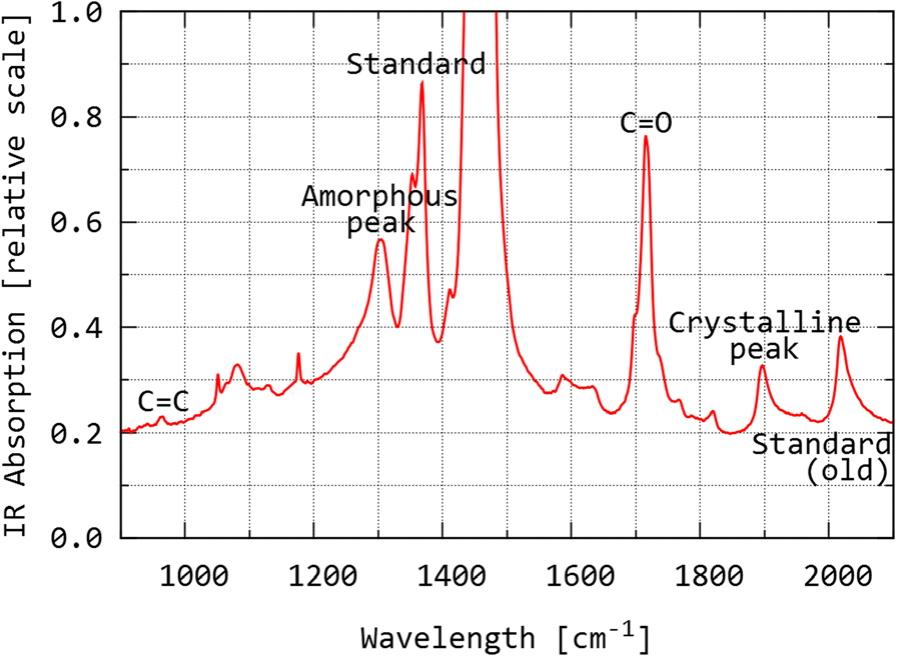
Part of IR spectrum from the 200 μm UHMWPE section with marked key peaks for calculation of the oxidation index (OI), trans-vinylene index (VI), and crystallinity index (CI).

In the first step of *determination of oxidative degradation* using IR spectroscopy, thin (2 mm) plates were machined from the UHMWPE liners under intensive cooling with distilled water. In the second step, perpendicular columns (2 mm × 2 mm × liner thickness) were cut from the plates. The IR spectrum was measured: (a) directly from the 2 mm columns (in the case of unused UHMWPE liners) or (b) from 200μm thin cross-sections, which were cut from the columns by a sledge microtome (in the case of explanted UHMWPE liners, listed in Table 1). The advantage of measuring thin, 200 μm sections lies in the fact that we can determine the parameters of the structure from various sites of the liner: at the inner surface, in the middle and at the outer surface. In the case of explanted liners, this fact is fundamental - oxidation may differ significantly at various distances from the surface. The measurement of IR spectra was performed using the Bruker IFS 55 FTIR spectrometer equipped with the DTGS detector. The spectra were measured with a resolution of 2 cm^-1^ and 128 scans were accumulated in order to decrease interference within the spectrum. Several samples were measured using the Thermo Nicolet 6700 FTIR spectrometer with the Nicolet Continuμm^TM^ FTIR microscope equipped with a MCT detector. With this setup, it was possible to measure IR spectra in samples reliably and rapidly along the whole cross-section with a step height of 100 μm. Consequently, we obtained a detailed profile of both oxidation index (OI), trans-vinylene index (VI) and crystallinity index (CI), i.e. the OI, VI and CI values as a function of distance from the inner surface of the UHMWPE liner [18]. The individual IR spectra of given sample were gradually recorded from square surfaces with edges of 100 μm, a resolution of 4 cm^-1^ and involving 8 scans each. Figure 1 shows a typical transmission IR spectrum measured from the 200 μm UHMWPE segment. The spectrum includes several bands (peaks) that correspond to the vibrations of certain chemical groups within molecules of UHMWPE (see the orange marks in Figure 1). Using the area-under-peak ratios, it is possible to calculate: (i) the oxidation index (OI; ratio of the C = O band area at 1715 cm^-1^ and the standard band at 1370 cm^-1^), (ii) the trans-vinylene index (VI; ratio of the C = C band at 965 cm^-1^ and the standard band at 1370 cm^-1^) and (iii) crystallinity index (CI; ratio of the crystalline and amorphous bands at 1897 cm^-1^ and 1303 cm^-1^, respectively). The values of OI and CI are proportional to oxidation damage and weight fraction of the crystalline phase, while VI may be used to estimate the radiation dose used during UHMWPE modification and/or sterilization [10,11,18].

*Determination of the concentration of free radicals* was measured by the electron spin resonance (ESR). The 2 mm columns were inserted into an ESR glass tube (maximum diameter 3 mm). The tube with the sample was measured by ESR spectrometer (Bruker ELEXYS E-540). The spectra were obtained at a microwave power of 6 mW using high-frequency magnetic modulation (100 kHz) with amplitude of 1G. The quality of a spectrometer cavity loading (Q) was around 4000. Double integration of these spectra can be used to determine the relative concentration of radicals while comparison with the spectrum of the calibrated standard can be used to determine the absolute concentration of radicals in the sample [11]. The absolute concentration of radicals in the sample is usually denoted as FRC (free radical concentration or residual radical concentration) [6]. The shapes of the peaks in the ESR spectra contain information about the structure of radicals.

*Microhardness* (MH) was measured by means of a microhardness tester (VMHT AUTO man; UHL) using Vickers method (square pyramid of diamond with angles between non-adjacent faces 136° is forced against flat surface of the specimen). For unused UHMWPE components, a minimum of 30 indents were made (3 smooth surfaces prepared by microtomy, 10 indents on each, load *F* = 50 gf; load time *t* = 6 s), average diagonal length (*d* = average of the two diagonals of the indent) was measured with a light microscope and the final MH value was calculated [19]:

(1) $$\;\mathrm{MH}\left[\mathrm{MPa}\right]\;=1.854\times F\left[N\right]/d^{2}\left[mm^{2}\right].$$

For explanted liners, the MH values were measured as a function of distance from the articulating surface. Each MH value at given distance from the surface represents an average from at least three measurements. Written informed consent was obtained from participants. The study does not include research on human subjects, human material and human data. Any personal data of the patient are not used. The study is based on research funded by a grant IGA NT12229-4/2011, which was assigned on the basis of this research project, which did not require approval of the ethics committee.

## Results and discussion

**Figure 2:**
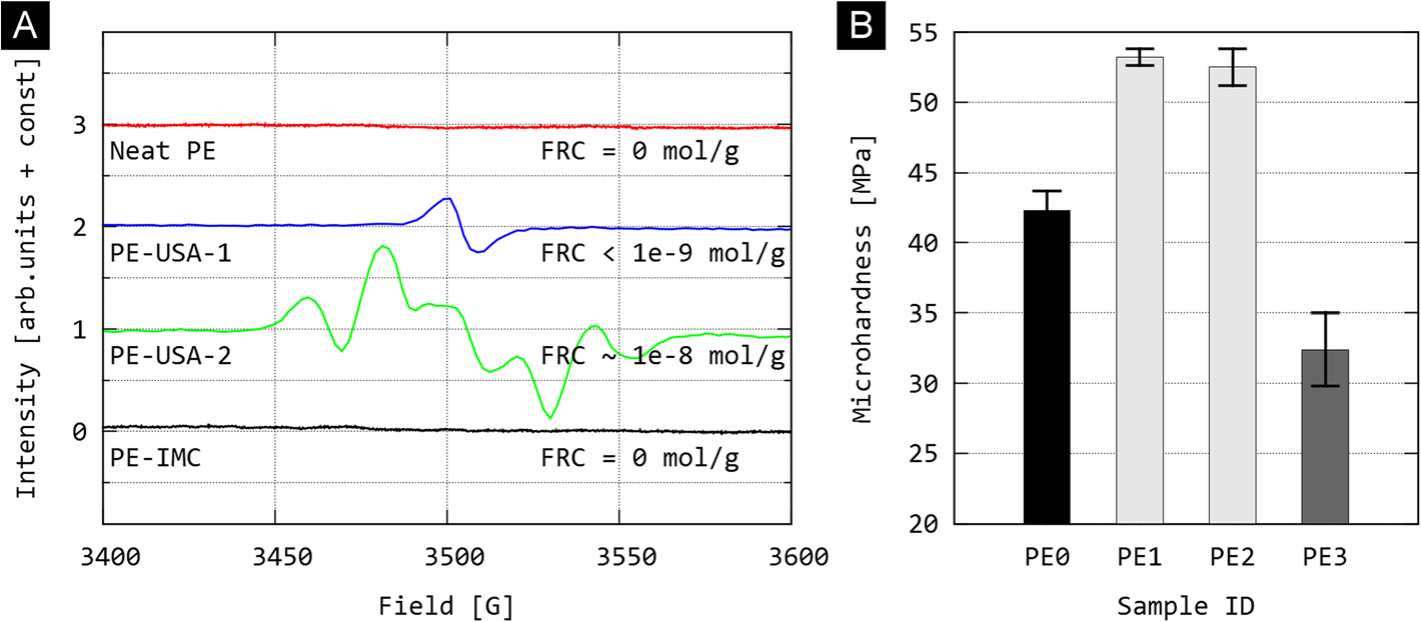
**ESR spectra (A) and MH values (B) of new, unused UHMWPE components PE0–PE3 (see also Table**2**).**

The results of measurements of new UHMWPE components are summarised in Table 2. When determining the oxidation indices (OI) and trans-vinylene indices (VI) from the IR measurements, the following facts must be considered: (i) If IR measurement was made from the 2 mm thick segments, it was necessary to use a standard peak of lower intensity (Figure 1, peak marked as Standard #2, wavenumber 2020 cm^-1^), because the preferred standard peak according to reference exhibited too high intensity or even overflow. (ii) Consequently, the OI and VI results recommended by the international standard [20], which are comparable with most data published today, must be attained by multiplying with the constant 0.354, which would lower the values in the Table 2.(iii) According to an overview of the available literature, if the UHMWPE component has an OI > 1 in a bearing region, the probability of joint replacement failure increases significantly, and if the OI is > 3 in this region, the material is so degraded that the failure of the joint replacement is merely a question of time [21,22]. As to the ESR measurements, these involved standard determination of free radicals (residual radicals from sterilization) that represent a risk from the aspect of further oxidative degradation. The residual radicals were detected only in the samples PE1 and PE2 (Figure 2A). The fact that the residual radicals increase oxidative degradation is documented in Table 2, while the connection between oxidative degradation and mechanical properties is illustrated in Figure 2B. Briefly, changes at molecular level (free-radical-induced oxidative degradation of polymer chains), change supermolecular structure (increase in crystallinity and lamellar thickness due to additional crystallization), which impacts also on local mechanical properties (such as microhardness, as demonstrated here). Detailed explanation of these effects was given in our previous studies [4,8].

**Table 2:** Results of IR and ESR analysis in new, unused UHMWPE joint replacement components

**Sample**	**IR**	**IR**	**ESR**
Designation	OI ()	VI ()	FRC mol/g
PE0	0.00	0.000	0
PE1	0.24	0.080	< 1e-9
PE2	0.11	0.044	~ 1e-8
PE3	0.03	0.064	0

**Figure 3:**
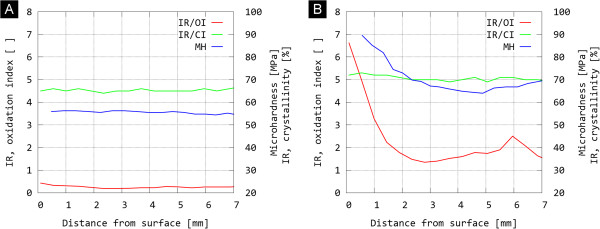
Typical oxidation index profiles, crystallinity profiles and microhardness profiles of (A) explanted UHMWPE liner with lower oxidation damage – sample L2 and (B) explanted UHMWPE liner with very high oxidation damage – sample L4.

The results of measurements of explanted UHMWPE liners (Table 1) are summarised in Table 3. In the case of explanted UHMWPE components, we evaluate oxidation profiles, which are values of oxidative damage as a function of distance from the inner surface. Typical oxidation profiles are depicted in Figure 3. At the same time, there is usually a difference between the oxidation profiles from the locations affected by wear (worn surfaces) and the locations not affected by wear (unworn surfaces).

**Table 3:** Results of IR and ESR measurements of explanted UHMWPE components

**Sample**	**Location of sample**	**Years in vivo**	**Oxidation indices, various sites**			**VI range**	**FRC (mol/g)**
			**OI inner**	**OI middle**	**OI outer**		
L1	Unworn	11	1.79	0.08	0.85	0.010–0.016	1.4e-10
	Worn		2.37	0.09	0.98		
L2	Unworn	20	0.43	0.19	0.28	0.014–0.020	5.0e-10
	Worn		1.52	0.22	0.89		
L3	Unworn	16	0.64	0.06	0.34	0.008–0.012	1.4e-10
	Worn		1.55	0.06	0.49		
L4	Unworn	10	6.62	1.35	2.50	0.012–0.018	5.2e-10
	Worn		2.14	1.52	2.34		

According to the weakest link theory, damage of the polymer is expected to occur in a bearing region at the locations with the highest oxidation index, i.e. at the most degraded locations. Thus it is suitable to characterise each explanted component using six oxidation index values: the maximum oxidation index near the inner surface (Table 3, OI inner); the maximal oxidation index in the middle of the material (Table 3, OI middle) and the maximal oxidation index in the vicinity of the outer surface (Table 3, OI outer). All three OI values (inner, middle, outer) are obtained for both locations (worn, unworn). The values of trans-vinylene index (VI) are quite constant within the experimental error throughout the whole explanted UHMWPE component (Table 3, VI). The free radical concentration (FRC) in the UHMWPE component was determined as the average from the location in the middle of the sample (Table 3, FRC).

Figure 3 illustrates the correlation among the structural changes at molecular level (oxidative damage), structural changes at supermolecular level (increase in crystallinity) and local mechanical properties (microhardness). For sake of brevity, we present just one typical case of UHMWPE liner with moderate oxidation damage (Figure 3A) and very high oxidation damage (Figure 3B). The local properties (oxidative degradation, crystallinity, microhardness) are mutually connected as follows: the increase in oxidative damage (oxidation index, proportional to polymer chain scissions), results in small but detectable increase in crystallinity (crystallinity index, proportional to overall crystallinity, which increases due to additional crystallization [4,8]) and microhardness (MH values are proportional to overall crystallinity [4,18,19]).

As for the new, unused UHMWPE components (Table 2), the samples measured using IR and ESR were taken from the middle of the studied component. Our decision was based on the fact that parallel experiments demonstrated that the unused components do not exhibit a significant difference between the surface and interior as far as the material structure and properties are concerned. This is due to the fact that the unused, appropriately sterilized, and barrier-packaged UHMWPE has not yet undergone the oxidative degradation, which principally progresses from the surface inwards. It is worth noting that the rate of oxidative degradation strongly depends on the type of UHMWPE and may occur not only during its storage (shelf life), when the material is exposed to oxygen from air, but also during the period when the polymer component is in a human body (in vivo), when the material is exposed to body fluids containing dissolved oxygen and other oxidising compounds [23]. The following facts follow from the aforementioned data. It has been confirmed that virgin polyethylene (PE0), which has not been modified by radiation or sterilised and has been stored correctly, does not show any measurable oxidative damage (proportional to the OI value) and no cross-linking (proportional to the VI value) as well as no risk from the aspect of long term oxidative degradation (proportional to the FRC value). From the aspect of oxidative damage (OI), practically all modified materials (PE1, PE2, PE3) were of sufficient quality, although the best properties were found for the last sample (PE3). As far as for the aspect of cross-linking, which may be approximately estimated from the vinylene index (VI), we may roughly arrange the studied types according to the degree of cross-linking (which is presumed to be proportional to the resistance to wear) as follows: PE1 > PE3 > PE2 > > PE0; it is worth noting that this is in perfect agreement with the data available from manufacturers, specified in the Experimental section. Concerning the aspect of free radical content, which represents a not-negligible risk of long-term in vivo oxidative degradation (i.e. the deterioration of mechanical properties after the implantation), the following series was obtained: PE0 = PE3 < PE1 < PE2.

As for the explanted UHMWPE components (Tables 1 and 3), typical profiles of oxidation index, crystallinity and microhardness are shown on Figure 3. Oxidation index usually exhibits maximum close to the articulating surface (Table 3: OI, inner), then decreases (Table 3: OI, middle), and shows second local maximum at the opposite surface (Table 3: OI, outer). The oxidation index correlated with joint replacement performance: the two samples with the highest OI values (L1, L4), exhibited the shortest lifetime in vivo. The trans-vinylene index of all the explanted components was comparable and basically independent on the distance from the implant surface, reaching values of around 0.015 (see Table 3). This corresponded to the fact that all liners were gamma sterilised with approximately the same dose of gamma radiation (conventionally 25–40 kGy). Analogously, the residual radical concentration in all four samples was the same within experimental error (we note that the concentrations are quite low, but detectable amounts of radicals survived in the polymer for >20 years). In general, the free radical concentration decreases with time. When comparing the FRC values of new and explanted UHMWPE components (compare Tables 2 and 3), it seems that the experimental data confirm the decrease in FRC with time. However, the measurements were performed independently, in several different experiments; a serious comparison would require concurrent measurements using absolutely the same standards in order to achieve higher accuracy.

When testing explanted polyethylene components, we must take into account not only the actual values of the of OI, VI, and FRC, but also the history of the component: modification and sterilization of UHMWPE liner, its shelf-life before the implantation, packaging, sterilization, and the oxidation damage and concentration of radicals, as well as the period during which the implant was present in vivo. At the same time, the patient activity and BMI (body mass index) plays an important role. Last but not the least, the surgical technique (selection of suitable joint replacement type and size, centring of the component, scratching of the component during the surgery [7]) may also influence the final lifetime of the artificial joint. There is a whole range of these factors and only analysis of a greater number of explanted components can yield some general conclusions. It is known from the professional literature that oxidative damage significantly reduces the mechanical properties of UHMWPE [15] and, consequently, the lifespan of joint replacements. These changes may fundamentally alter the quality and material properties of the components. In clinical practice this means that one cannot deduce the quality and lifespan of the articular liner based only on its initial mechanical properties [12]. An orthopaedic surgeon should take into account also the long-term oxidation stability (inversely proportional to FRC). The value of FRC (i.e. the concentration of residual radicals from the irradiation during the material processing) should be zero, i.e. below the ESR detection limit or the product should be stabilized with vitamin E [1,2,4,11,17].

Within the results achieved in this study, it is interesting to follow the development of UHMWPE components produced by Beznoska company (Beznoska Kladno, Czech Republic). Our study includes three Beznoska samples: (i) Beznoska UHMWPE liner from 1991 – sample L3 in Tables 1 and 3, (ii) Beznoska UHMWPE liner from 1996 – sample L4 in Tables 1 and 3 and (iii) unused Beznoska UHMWPE liner from 2007 – sample PE3 in Table 2. According to the information available to the authors of this study, the first UHMWPE component from year 1991 (L3) was produced by traditional technique (no special modification of polymer, gamma sterilization in low oxygen atmosphere, barrier packaging). This technology was relatively reliable and, as a result, the sample exhibited quite low oxidation damage even after 16 years in vivo (Table 3). The second studied Beznoska UHMWPE component from 1996 (L4) suffered from an unspecified polymer processing mistake, which was not recognized at that time. Nevertheless, our measurement revealed severe oxidation damage of the 1996 component in comparison with all other samples (cf. values of OI in Tables 2 and 3). Also searching in clinical data revealed a number of other Beznoska liners implanted in 1996, which failed shortly after the surgery. The third studied Beznoska UHMWPE component from 2007 showed, in comparison with the other liners, the lowest oxidation damage (the lowest OI value), comparable level of radiation dose absorbed during the processing (average VI value), and the lowest risk of long-term oxidative degradation (zero FRC value).

## Conclusion

In total joint replacements, the manufacturers use various types of UHMWPE. The structure and properties of the UHMWPE liners may differ considerably. The orthopaedic surgeons usually do not have access to objective information concerning the particular UHMWPE liner quality.

In this contribution we have demonstrated that the IR and ESR can provide the surgeons with valuable pieces of information concerning the UHMWPE quality. The methods are relatively fast and yield the following data: (i) Oxidation index (OI), which is a measure of oxidative degradation, (ii) trans-vinylene index (VI), which is proportional to the radiation dose absorbed by UHMWPE during the material processing, (iii) crystallinity index (CI), which correlates with weight fraction of crystalline phase, and (iv) free radical concentration (FRC), which is associated with the long-term oxidative stability. The IR and ESR measurements may be supplemented by microhardness testing (MH) that verifies the impact of structural changes to local mechanical properties.

The extent of oxidative degradation (OI) is the key parameter: the more degraded UHMWPE, the worse from the point of view of its future lifetime [6]. It follows from the fact that the oxidative degradation of UHMWPE worsens all mechanical properties including the crucial property for given application – wear resistance [1,4,7,9,10,15]. The information about radiation dose absorbed during the material processing (VI) informs us if the material was crosslinked and/or sterilized by gamma irradiation. The amount of residual radicals (FRC) should be zero in modern UHMWPE liners, because their presence is risky from the point of view of long-term oxidation stability of the replacement.

We conclude that the independently measured parameters of a given polyethylene component (OI, VI and FRC, which can be supplemented with CI and MH for more detailed analysis) may help an orthopaedic surgeon choose the best UHMWPE joint replacement component and thus radically avoid early joint replacement failure due to inferior polyethylene quality that we have had occasionally observed in recent years. This report also demonstrated that the IR and ESR methods are applicable not only for testing new UHMWPE components from various manufacturers, but also for analysis of total joint replacement failures based on the explanted failed UHMWPE components.

## Competing interests

Institute of Macromolecular Chemistry, Academy of Sciences of the Czech Republic, Heyrovsky Sq. 2, 162 06 Prague 6, Czech Republic is holder of patent (Czech patent CZ 297700 (2007)) of production of one of the tested samples (PE3).

## Authors’ contributions

PF is an orthopedic surgeon, dealing with issues of lifetime of joint replacements. Participated in the study design, sampling, recording, collecting anamnestic data and is a major author of the text of this study. DP is a significant orthopedic surgeon, dealing with issues of lifelong lifetime of joint replacements. Team leader for orthopedic clinic and supervisor of the main author. He participated in the sampling, interpretation of data and creation of the final text of the study. MS is a worldwide known chemist involved in the macromolecular structure of polymers. Author of many worldwide recognized works. Head of research team for the Institute of Macromolecular Chemistry, Academy of Sciences of the Czech Republic. One of the authors of the material part of study, coauthor of design of the study and sample testing coordinator. MN is a chemist involved in the macromolecular structure of polymers. She was responsible for working with samples, testing coordination and participated in the interpretation of data in the final text of the study. TV deals with the material characteristics of polymers. She measured microhardness of the tested samples and interpretation of the data. JD deals with the material characteristics. He measured oxidative damage by using infrared spectroscopy and interpretation of the data. JP is a material chemist. He performed the measurement of the concentration of free radicals using by electron spin resonance method and interpretation of the data. All authors read and approved the final manuscript.

## Pre-publication history

The pre-publication history for this paper can be accessed here:

http://www.biomedcentral.com/1471-2474/15/109/prepub
